# Petroleum Hydrocarbon-Degrading Bacteria for the Remediation of Oil Pollution Under Aerobic Conditions: A Perspective Analysis

**DOI:** 10.3389/fmicb.2018.02885

**Published:** 2018-12-03

**Authors:** Xingjian Xu, Wenming Liu, Shuhua Tian, Wei Wang, Qige Qi, Pan Jiang, Xinmei Gao, Fengjiao Li, Haiyan Li, Hongwen Yu

**Affiliations:** ^1^Northeast Institute of Geography and Agroecology, Chinese Academy of Sciences, Changchun, China; ^2^Hinggan League Academy of Agriculture and Animal Husbandry, Ulanhot, China; ^3^School of Life Science and Technology, Changchun University of Science and Technology, Changchun, China

**Keywords:** petroleum hydrocarbon-degrading bacteria, petroleum oil, bioremediation, bacterial consortia, environmental factors, enzymes

## Abstract

With the sharp increase in population and modernization of society, environmental pollution resulting from petroleum hydrocarbons has increased, resulting in an urgent need for remediation. Petroleum hydrocarbon-degrading bacteria are ubiquitous in nature and can utilize these compounds as sources of carbon and energy. Bacteria displaying such capabilities are often exploited for the bioremediation of petroleum oil-contaminated environments. Recently, microbial remediation technology has developed rapidly and achieved major gains. However, this technology is not omnipotent. It is affected by many environmental factors that hinder its practical application, limiting the large-scale application of the technology. This paper provides an overview of the recent literature referring to the usage of bacteria as biodegraders, discusses barriers regarding the implementation of this microbial technology, and provides suggestions for further developments.

## Introduction

Petroleum oil is an important strategic resource for which all countries compete fiercely (Sun, 2009). Indeed, anthropogenic activity is reliant on oil to meet its energy demands, which causes the petrochemical industry to flourish. However, petroleum use results in environmental deterioration (Xue et al., 2015). During petroleum production, storage and transportation, refining and processing, as well as spills and discharges of petroleum hydrocarbons often occur as a result of blowout accidents during oilfield development, leakage from oil pipelines and storage tanks, oil tanker and tanker leakage accidents, oil well waxing, and during overhauls of refineries and petrochemical production equipment (Chaerun et al., 2004; Chen et al., 2015; Wang C. et al., 2018). Large spills should be recycled or eliminated to as great a degree as possible, but in some cases it is difficult to recover the spilled materials, resulting in its remaining in the affected area, and posing persistent risks to the environment.

Accordingly, there is a constant threat of contamination wherever oil is exploited when coupled with an insufficient ability to deal with oil-contaminated environments, especially in extreme or unique environments such as polar regions, deep sea areas, deserts, and wetlands. Although oil pollution is difficult to treat, petroleum hydrocarbon-degrading bacteria have evolved as a result of existing in close proximity to naturally occurring petroleum hydrocarbons in the environment. Such organisms are candidates for the treatment of oil pollutants (Margesin et al., 2003; Ron and Rosenberg, 2014; Lea-Smith et al., 2015). Therefore, bacteria have been screened and utilized to degrade waste products produced by the food, agricultural, chemical and pharmaceutical industries. In recent years, the use of bacteria to deal with environmental pollutants has become a promising technology because of its low cost and eco-friendly nature (Guerra et al., 2018). The continuous development and improvement of microbial remediation technology has also provided a new method for the remediation of petroleum hydrocarbon pollution, which has attracted much attention (Dombrowski et al., 2016; Dvořák et al., 2017). The purpose of this review article is to provide some suggestions for the future development of bacterial remediation of petroleum hydrocarbons on the basis of previously published studies related to new advances in the area of bacterial remediation of petroleum hydrocarbons.

## Petroleum Hydrocarbon-Degrading Bacteria

Most petroleum hydrocarbons encountered in the environment are ultimately degraded or metabolized by indigenous bacteria because of their energetic and carbon needs for growth and reproduction, as well as the requirement to relieve physiological stress caused by the presence of petroleum hydrocarbons in the microbial bulk environment (Hazen et al., 2010; Kleindienst et al., 2015a). The development of microbial biotechnology and high-throughput sequencing technology, such as microfluidic techniques (Jiang et al., 2016; Guerra et al., 2018), is beneficial for screening and identifying functional microorganisms from petroleum hydrocarbon-contaminated environments. Indeed, many studies have revealed that there is a large number of hydrocarbon-degrading bacteria in oil-rich environments, such as oil spill areas and oil reservoirs (Hazen et al., 2010; Yang et al., 2015), and that their abundance and quantity are closely related to the types of petroleum hydrocarbons and the surrounding environmental factors (Fuentes et al., 2015; Varjani and Gnansounou, 2017).

Many normal and extreme bacterial species have been isolated and utilized as biodegraders for dealing with petroleum hydrocarbons. The degradation pathways of a variety of petroleum hydrocarbons (e.g., aliphatics and polyaromatics) have been shown to employ oxidizing reactions; however, these pathways differ greatly because of the specific oxygenases found in different bacterial species. For instance, some bacteria can metabolize specific alkanes, while others break down aromatic or resin fractions of hydrocarbons. This phenomenon is related to the chemical structure of petroleum hydrocarbon components. Petroleum hydrocarbon-degrading bacteria and the type of petroleum components they degrade are listed in Table 1. Recent studies have identified bacteria from more than 79 genera that are capable of degrading petroleum hydrocarbons (Tremblay et al., 2017); several of these bacteria such as *Achromobacter*, *Acinetobacter*, *Alkanindiges*, *Alteromonas*, *Arthrobacter*, *Burkholderia*, *Dietzia*, *Enterobacter*, *Kocuria*, *Marinobacter*, *Mycobacterium*, *Pandoraea*, *Pseudomonas*, *Staphylococcus*, *Streptobacillus*, *Streptococcus*, and *Rhodococcus* have been found to play vital roles in petroleum hydrocarbon degradation (Margesin et al., 2003; Chaerun et al., 2004; Jin et al., 2012; Nie et al., 2014; Varjani and Upasani, 2016; Sarkar et al., 2017; Varjani, 2017; Xu et al., 2017). Interestingly, “conditionally rare taxa” in soil, such as *Alkanindiges* sp., have been reported to exhibit rare-to-dominant bacterial shifts that are strongly affected by environmental constrains such as diesel pollution (Fuentes et al., 2015). Similarly, some obligate hydrocarbonoclastic bacteria (OHCB), including *Alcanivorax*, *Marinobacter*, *Thallassolituus*, *Cycloclasticus*, *Oleispira* and a few others (the OHCB), showed a low abundance or undetectable status before pollution, but were found to be dominant after petroleum oil contamination (Yakimov et al., 2007). These phenomena suggest that these microorganisms are crucial to the degradation of petroleum hydrocarbons, and that they significantly influence the transformation and fate of petroleum hydrocarbons in the environment. Although some bacteria have been reported to have a broad spectrum of petroleum hydrocarbon degradation ability, *Dietzia* sp. DQ12-45-1b utilizes *n*-alkanes (C6–C40) and other compounds as the sole carbon sources (Wang et al., 2011) and *Achromobacter xylosoxidans* DN002 works well on a variety of monoaromatic and polyaromatic hydrocarbons (Ma et al., 2015), almost no bacteria can degrade the entire petroleum hydrocarbon fraction. Indeed, most bacteria can only effectively degrade or utilize certain petroleum hydrocarbon components, while others are completely unavailable (Chaerun et al., 2004; Varjani, 2017). This can be attributed to the fact that different indigenous bacteria have different catalytic enzymes; thus, their roles in oil-contaminated sites also vary widely. This also implies that the remediation of petroleum hydrocarbon contamination requires the joint action of multiple functional bacteria to achieve the best environmental purification effect (Dombrowski et al., 2016). Based on this view, Varjani et al. (2015) constructed a halotolerant Hydrocarbon Utilizing Bacterial Consortium (HUBC) consisting of the bacterial isolates *Ochrobactrum* sp., *Stenotrophomonas maltophilia* and *Pseudomonas aeruginosa* that was found to be good at degrading crude oil (3% v/v), with a degradation percentage as high as 83.49%. Tao et al. (2017) utilized a defined co-culture of an indigenous bacterial consortium and exogenous *Bacillus subtilis* to effectively accelerate the degradation of crude oil. Wang C. et al. (2018) found that an aboriginal bacterial consortium based on the Penglai 19-3 oil spill accident (China) had higher oil degradation efficiency compared to individual bacteria and demonstrated that this indigenous consortium had the potential for bioremediating crude oil dispersed in the marine ecosystem. A field study showed that bioaugmentation with an artificial consortium containing *Aeromonas hydrophila*, *Alcaligenes xylosoxidans*, *Gordonia* sp., *Pseudomonas fluorescens*, *Pseudomonas putida*, *Rhodococcus equi*, *S. maltophilia*, and *Xanthomonas* sp. contributed to high biodegradation efficiency (89%) in a 365-day treatment of diesel oil-contaminated soil (Szulc et al., 2014). Taken together, these studies indicate that improving the biodegradation potential via the application of bacterial consortia possessing multiple catabolic genes is a reasonable and feasible strategy for accelerating the removal efficiency of petroleum hydrocarbons from contaminated environments.

**Table 1:** Petroleum hydrocarbon-degrading bacteria and their preferred degradation substrates.

Petroleum hydrocarbon components	Bacterial species	Main degradation profile	Reference
Aliphatics	*Dietzia* sp.	*n*- alkanes (C6-C40)	Wang et al., 2011
	*Pseudomonas* sp.	*n*-alkanes (C14–C30)	Sugiura et al., 1997
	*Oleispira antarctica*	*n*-alkanes (C10–C18)	Yakimov et al., 2003
	*Rhodococcus ruber*	*n*-alkanes (C13–C17)	Zhukov et al., 2007
	*Geobacillus thermodenitrifican*	*n*-alkanes (C15–C36)	Abbasian et al., 2015
	*Rhodococcus* sp.	Cyclohexane	Lee and Cho, 2008
	*Alcanivorax* sp.	*n*-alkanes and branched alkanes	Hara et al., 2003
	*Gordonia sihwensis*	Branched and normal alkanes	Brown et al., 2016
Aromatics	*Achromobacter xylosoxidans*	Mono-/polyaromatics	Ma et al., 2015
	*Aeribacillus pallidus*	Mono-/polyaromatics	Mnif et al., 2014
	*Mycobacterium cosmeticum*	Monoaromatics	Zhang et al., 2013
	*Pseudomonas aeruginosa*	Monoaromatics	Mukherjee et al., 2010
	*Cycloclasticus*	Polyaromatics	Kasai et al., 2002
	*Neptunomonas naphthovoran*	Polyaromatics	Hedlund et al., 1999
	*Bacillus Licheniformis* *Bacillus Mojavensis*	Polyaromatics	Eskandari et al., 2017
	*Sphingomonas, Sphingobium* and *Novosphingobium*	Polyaromatics	Ghosal et al., 2016
Resins and asphaltenes	*Pseudomonas* sp.	Resins	Venkateswaran et al., 1995
	*Pseudomonas* spp., *Bacillus* sp.	Asphaltenes	Tavassoli et al., 2012
	*Citrobacter* sp., *Enterobacter* sp., *Staphylococcus* sp., *Lysinibacillus* sp. *Bacillus* sp., *Pseudomonas* sp.	Asphaltenes	Jahromi et al., 2014


**FIGURE 1:**
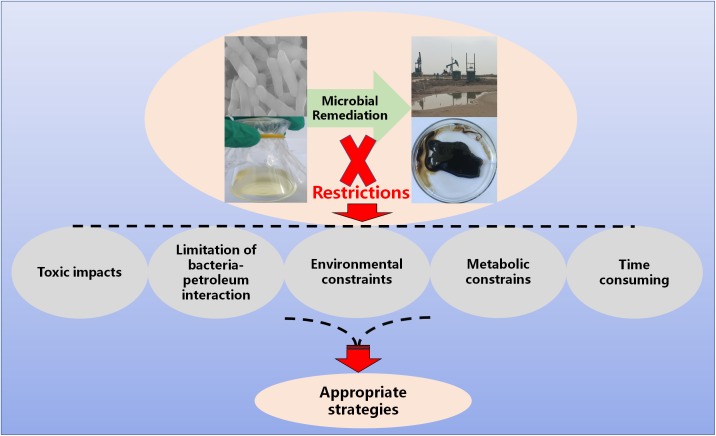
Restrictions of microbial remediation.

*Some petroleum hydrocarbon-degrading strains have been studied as model organisms to further reveal the mode of action of bacteria in degrading petroleum hydrocarbons, degradation pathways, molecular mechanisms, etc. Moreover, it has been known for some time that the use of bacteria to degrade petroleum hydrocarbons is not always successful and is affected by many factors, including the toxic effects of petroleum hydrocarbons on bacteria, the bioavailability of petroleum hydrocarbons, environmental constraints, metabolic restriction and long remediation periods (Head et al., 2006; Figure 1).*

## Toxic Impact of Petroleum Hydrocarbons

The harm that oil pollution causes to the ecological environment is well known (Sikkema et al., 1995). For example, the Deep Water Horizon oil spill accident in the Gulf of Mexico produced a profound impact on the economy and environmental safety, which is still the focus of people’s attention (Xue et al., 2015). Although people are becoming increasingly concerned about the toxic effects of oil pollution on humans and animals in affected areas, (Díez et al., 2007; Mason et al., 2012), the strong toxic impacts of petroleum hydrocarbons on affected microbial communities are often overlooked (Rivers et al., 2013; Overholt et al., 2015). Labud et al. (2007) reported that petroleum hydrocarbons inhibited microbial biomass, and that the greatest negative effects were observed in the gasoline-polluted sandy soil. In diesel exposure experiments, researchers found that the primary effects of diesel fuel toxicity were reductions in species richness, evenness and phylogenetic diversity, with the resulting community being heavily dominated by a few species, principally *Pseudomonas*. Moreover, they found that the decline in richness and phylogenetic diversity was linked to the disruption of the nitrogen cycle, with species and functional genes involved in nitrification being significantly reduced (van Dorst et al., 2014). Cerniglia et al. (1983) investigated the toxicity of naphthalene, 1-methylnaphthalene, and 2-methylnaphthalene as well as their oxygenated derivatives to bacterial cells of *Agmenellum quadruplicatum*, and found that these compounds produced no significant inhibitory effects on bacterial growth. However, the phenolic and quinonic naphthalene derivatives inhibited bacterial growth. This could be explained by phenols and quinones with higher solubility, enhancing the mass transfer of molecules to bacterial cells, resulting in higher toxic effects than the former compounds. Several studies have also reported that certain metabolic intermediates with relatively high solubility produced from the degradation of petroleum hydrocarbons by bacteria may have higher cytotoxicity than the parent molecules and therefore damage the bacteria (Hou et al., 2018). However, indigenous bacteria form very large aggregates, and each species has its own function. Accordingly, while some bacteria that are sensitive to petroleum hydrocarbons are greatly inhibited upon exposure to petroleum hydrocarbons, others that can efficiently degrade petroleum hydrocarbons, as well as bacteria that can take advantage of cytotoxic intermediate metabolites, will flourish. However, clean-up of petroleum oil pollutants by relying on the strength of these indigenous microorganisms alone will take a long time; therefore, it is necessary to develop intervention measures to speed the process up.

## Restriction of Physical Contact Between Bacteria and Petroleum Hydrocarbons

Due to the hydrophobicities and low water solubilities of most petroleum hydrocarbons, the biodegradation rate is generally limited in the environment. This is because the first step in the degradation process of petroleum oil often requires the participation of bacterial membrane-bound oxygenases, which require direct and effective contact between bacterial cells and petroleum hydrocarbon substrates. The primary factors restricting the biodegradation efficiency of petroleum hydrocarbons are as follows: (1) limited bioavailability of petroleum hydrocarbons to bacteria, and (2) the fact that bacterial cell contact with hydrocarbon substrates is a requirement before introduction of molecular oxygen into molecules by the functional oxygenases (Vasileva-Tonkova et al., 2008; Hua and Wang, 2014). However, bacteria have evolved countermeasures against petroleum contaminants, such as improving the adhesion ability of cells by altering their surface components and secreting bioemulsifier to enhance their access to target hydrocarbon substrates. Bacteria with such functions are often screened for use as environmental remediation agents, accelerating the removal of petroleum hydrocarbon pollutants from the environment (Kaczorek et al., 2012; Krasowska and Sigler, 2014).

**FIGURE 2:**
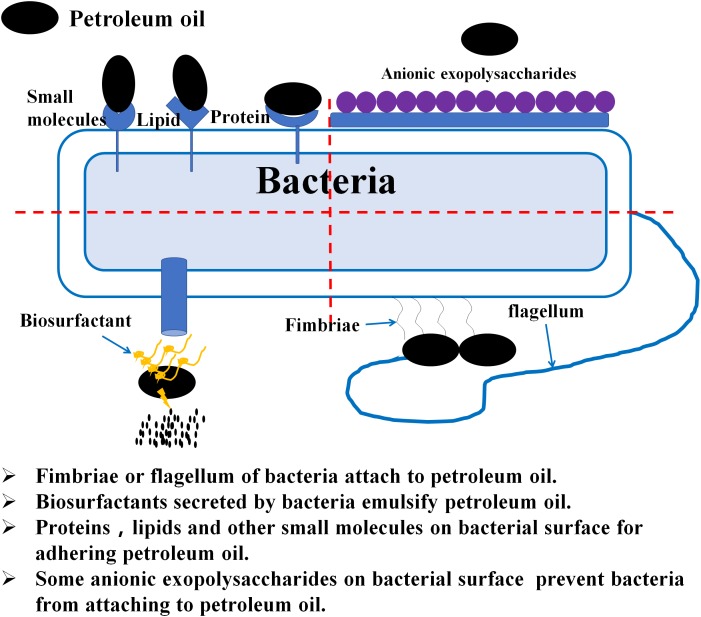
Schematic diagram of the physical contact between bacteria and petroleum hydrocarbons.

Bacterial surface properties are essential to the effective biodegradation of hydrophobic hydrocarbon substrates (Figure 2) and their adhesion mechanisms are of great importance (Zhang et al., 2015). Ron and Rosenberg (2014) found that adherence of hydrophobic pollutants to bacterial cells is mainly related to hydrophobic fimbriae, fibrils, outer-membrane proteins and lipids, as well as certain small molecules present in cell surfaces such as gramicidin S and prodigiosin. Fimbriae present on bacterial surfaces were confirmed to be necessary for the growth of *Acinetobacter* sp. RAG-1, with C_16_ alkane as the carbon source and beneficial to bacterial adherence, assimilation hydrophobic substrates and their metabolic activity (Rosenberg and Rosenberg, 1985). Nevertheless, bacterial capsules and several anionic exopolysaccharides produce inhibitory effects on hydrocarbon substrate adhesion. For example, *Bacillus licheniformis* decreases cell surface hydrophobicity in response to exposure to organic solvents and has little affinity for toxic organic compounds (Torres et al., 2011). Although bacterial adherence can enhance the biodegradation of hydrophobic hydrocarbons, it is not necessary to attach bacterial cells to targeted substrates (Abbasnezhad et al., 2011). This is because, in some instances, bacteria with high surface hydrophobicity are easily aggregated and form biofilms, thereby producing potential risks such as diseases (Doyle, 2000). Indeed, not only hydrophobic bacteria can biodegrade hydrophobic pollutants; several solvent-resistant hydrophilic bacteria are also capable of metabolizing such pollutants (Heipieper et al., 2007), which may be because of the modification of lipopolysaccharides or porines of the outer membrane of the bacterial surface (Krasowska and Sigler, 2014). Megharaj et al. (2011) also reported that the solvent-resistant bacteria were first to colonize and dominate for the removal of pollutants. Therefore, the use of hydrophilic microorganisms to treat hydrocarbon pollutants seems to be more advantageous than hydrophobic microorganisms (Obuekwe et al., 2009).

To enhance the bioavailability of petroleum hydrocarbons, one promising approach is the application of surfactants (Kleindienst et al., 2015a), which may enhance dissolution or desorption rates leading to the solubilization or emulsification of petroleum hydrocarbon pollutants (Varjani and Upasani, 2017). Chen et al. (2007) found that the adherence of *Bacillus* sp. DQ02 to hydrocarbon increased 44% in the presence of rhamnolipids and that the degradation of *n*-hexadecane increased 11.6% compared to treatment in the absence of rhamnolipids. However, some surfactants, such as Corexit 9500, were reported to exhibit adverse impacts on oil-degrading bacteria (Kleindienst et al., 2015b) because of toxicity of the surfactant toward bacteria or as a result of competition of the surfactant with hydrocarbon substrates (Laha and Luthy, 1991; Liu et al., 2016). In view of this, the selection of appropriate surfactants is of great importance for pollution remediation and the prevention of secondary pollution. Bioemulsifier-producing bacteria, which have attracted much attention, generally have the following two physiological aspects: (1) the ability to enhance the complexation and solubilization of non-polar substrates, thereby promoting the bioavailability of substrates, and (2) the ability to improve affinity between cell surfaces and oil-water interfaces through metabolism, promoting deformation of the oil-water interface film (Hou et al., 2018). Ayed et al. (2015) reported that the biosurfactant produced by *Bacillus amyloliquefaciens* An6 was an alternative to chemically synthesized surfactants since it showed high solubilization efficiency toward diesel oil (71.54% at 1 g/L) that was better than SDS and Tween 80 and could enhance the diesel oil degradation efficiency of the An6 strain. However, not all the biosurfactants produced by bioemulsifier-producing bacteria can effectively enhance the degradation rate of pollutants (Inakollu et al., 2004). Indeed, whether various biosurfactants stimulate or inhibit the bioremediation of pollutants is dependent on the physico-chemical properties of the surfactants, types of pollutants and physiological characteristics of the functional microorganisms (Hua and Wang, 2014). Therefore, it is necessary to establish a database of petroleum hydrocarbon pollutants and bioemulsifier-producing bacteria which is conducive to the targeted selection of suitable bacteria to treat with petroleum hydrocarbons.

## Environmental Constraints

Many environmental factors such as temperature, nutrients, electron acceptors and substrates play vital roles in bioremediation and influence biodegradation reactions (Varjani and Upasani, 2017). This is why most researchers have found that many petroleum hydrocarbon-degrading bacteria can achieve excellent results during degradation of petroleum hydrocarbons under laboratory conditions yet exhibit dissatisfactory results in field-scale tests (Head et al., 2006). The bacterial strains *Acinetobacter* sp. JLS1 and *P. aeruginosa* JLC1, isolated from Momoge wetlands in Jilin Province, China, showed different sensitivity to temperature during the biodegradation process of C_16_ alkane, suggesting that temperature strongly affected biodegradation efficiency (Li et al., 2017; Xu et al., 2017). In a laboratory study, the petroleum hydrocarbons phenanthrene and dibenzothiophenes were well degraded, but similar degradation effects did not occur in a field experiment, which could be attributed to the temperature range during the study (Röling et al., 2002, 2004). Indeed, temperature can affect bacterial growth and metabolism, the soil matrix and the mode of occurrence of pollutants, thereby indirectly affecting biodegradation efficiency (Abed et al., 2015). It is well known that the growth of bacteria requires sufficient carbon, hydrogen, oxygen, nitrogen, sulfur, phosphorus, and various trace elements. However, the main components of petroleum hydrocarbons are only carbon and hydrogen, therefore the environment must have enough other nutrient elements to ensure growth of bacterial degraders. It is estimated that approximately 150 g of nitrogen and 30 g of phosphorous are consumed to convert 1 kg of hydrocarbons in bacterial cells (Ron and Rosenberg, 2014). Extensive laboratory and field studies have been devoted to solving this problem. The addition of fertilizers containing bioavailable nitrogen and phosphorus has been successfully applied to stimulate petroleum oil biodegradation on a number of different shorelines and sandy beaches (Röling et al., 2002; Hazen et al., 2016). Soluble and non-soluble nutrients suffer from problems in the actual remediation, leading to low bioremediation efficiencies (Ron and Rosenberg, 2014). Researchers have found that using nitrogen-fixing hydrocarbon-degrading bacteria to improve the bioremediation efficiency was another good strategy instead of providing nitrogen sources (Thavasi et al., 2006). For aerobic degradation processes, using oxygen as an electron acceptor is quite important, but it is usually not adequate in petroleum oil-contaminated environments because of the limited air permeability. Gogoi et al. (2003) reported that up to 75% of the hydrocarbon contaminants were degraded within 1 year in field tests by controlling and regulating aeration. However, providing a sufficient oxygen supply to stimulate the bioremediation of petroleum pollutants in the environment is rather expensive and not feasible. Hence, the application of bulking agents such as saw dust into the soil to increase permeability or other electron acceptors (NO_3_^-^, Fe^3+^, or Mn^2+^) into anoxic environments to stimulate anaerobic microorganisms is often more economical than oxygen supplementation (Zedelius et al., 2011; Brown et al., 2017).

## Metabolic Restriction

The ability to biodegrade petroleum oil is associated with the concentration and composition of hydrocarbons. Extremely high levels of petroleum hydrocarbons strongly inhibit bacterial growth, resulting in poor biodegradation efficiency and even death of the bacteria (Ma et al., 2015). As reported by Varjani (2017), the order of biodegradability of hydrocarbons is as follows: linear alkanes > branched alkanes > low molecular weight alkyl aromatics > monoaromatics > cyclic alkanes > polyaromatics > asphaltenes. This is related to the physico-chemical properties of the substrate and its bioavailability, which affect the contact, transport and transformation of bacteria to hydrocarbon substrates (Varjani and Upasani, 2016). The vast majority of indoor studies are focused on the degradation of a single substrate, but in nature the components of petroleum hydrocarbon pollutants are extremely complex. Accordingly, it is difficult to reproduce laboratory results in practical applications. For example, *Pseudomonas putida* F1 can efficiently mineralize benzene, toluene and phenol. While in the substrate mixtures, toluene and benzene enhance the biodegradation of phenol; however, phenol inhibits the biodegradation of benzene and toluene (Abuhamed et al., 2004).

The key components of bacterial degradation of petroleum hydrocarbons are various specific enzymes (Wasmund et al., 2009; Varjani, 2017). For example, the enzymes alkane 1-monooxygenase, alcohol dehydrogenase, cyclohexanol-dehydrogenase, methane monooxygenase and cyclohexanone 1,2 monooxygenase are involved in degradation of alkanes, while naphthalene 1,2-dioxygenase ferredoxin reductase component, *cis-*2,3-dihydrobiphenyl-2,3-diol dehydrogenase and salicylaldehyde dehydrogenase are associated with naphthalene degradation and benzene dioxygenase, toluene dioxygenase and ethylbenzene dioxygenase work on other petroleum hydrocarbons (Bacosa et al., 2018).

Many isolated bacteria possess the ability to mineralize chemically simple petroleum hydrocarbons completely, such as linear alkanes, as long as these bacteria possess all of the enzymes for the targeted substrate (Head et al., 2006; Seth-Smith, 2010; Margesin et al., 2013). However, few bacteria can completely mineralize complex compounds such as resins and asphaltenes because of the lack of some enzymes (Varjani, 2017). The advantages of microbial communities are presented because there are a variety of catabolic genes in a bacterial consortium, and the synergistic effects of these genes are beneficial to achieving the purification of pollutants (Gurav et al., 2017). A bacterial consortium composed of five culturable bacteria has been constructed by Wanapaisan et al. (2018). Researchers found that these five bacteria showed synergistic pyrene degradation due to the following aspects: (1) The *Bacillus* strain enhanced the bioavailability of the pyrene by producing biosurfactant, (2) two *Mycobacterium* strains contributed to the initiation of pyrene degradation, and (3) *Novosphingobium* and *Ochrobactrum* efficiently degraded the intermediates of pyrene. Given the complexity of the petroleum components, construction of the minimal functional bacterial consortium or genetic engineering bacteria for bioremediation of petroleum oil has become a trend in this field (Dvořák et al., 2017). However, the stability of the community and the safety of the engineered bacteria are additional problems that must be overcome.

## Time Consuming

The core element of bioremediation is functional microorganisms that need sufficient nutrients and suitable environmental conditions. In general, petroleum oil hydrocarbons are not necessary substrates for hydrocarbon-degrading bacteria, and they utilize these compounds as alternative carbon and energy sources, especially in the absence of their preferable substrates. The function of hydrocarbon-degrading bacteria mainly depends on their hydrocarbon-degrading enzymes, the expression and activity of which are closely related to the physiological activity of bacteria (Mukherjee et al., 2017; Song et al., 2017). Sufficient time is needed to synthesize hydrocarbon-degrading enzymes because of the requirements of bacterial growth and synthetic metabolism. Although some bacteria have been reported to mineralize petroleum hydrocarbons completely within several days, or even less than 1 day under culture conditions, the degradation efficiency of these bacteria makes it difficult to meet the expected effects in practical usage (Chen et al., 2017; Zheng et al., 2018). The complex combination of various biological and abiotic factors limits the function of petroleum hydrocarbon-degrading bacteria in many ways (Zhao et al., 2017; Wang Y. et al., 2018). The degradation rate of petroleum hydrocarbon in the actual environment is the result of these factors acting on the petroleum hydrocarbon-degradation bacteria, which has led to most microbial remediation technologies taking a long time, especially when compared to physico-chemical remediation techniques. For instance, Fida et al. (2017) found that the green fluorescent protein (GFP) tagged variant of *Novosphingobium* sp. strain LH128 showed a dramatic decrease in colony forming units (CFU) within 4 h and entered a viable but nonculturable (VBNC)-like state upon inoculation into phenanthrene spiked soil based on physiological and transcriptome analysis, which could be related to the response to environmental stimuli in the soil by expression of stress protective mechanisms. This disadvantage makes it almost impossible to do anything when dealing with emergency pollution incidents because bioremediation will not remove contaminants as soon as the contamination occurs, but rather requires sufficient time to be achieved. In addition, there is no time to screen for indigenous bacteria or flora in contaminated accident zones, and the application of exogenous bacteria requires scientific assessment, government approval, etc., all of which will consume time (Ivshina et al., 2015). However, microbial remediation technology plays an irreplaceable role in ecological security when dealing with petroleum hydrocarbon-polluted environments due to its low cost, positive effect, little environmental influence and lack of secondary pollution (Dvořák et al., 2017). Moreover, petroleum hydrocarbons are completely mineralized into carbon dioxide and water under the action of various microbes, although bioremediation is time-consuming. Hence, to effectively reduce the microbial remediation period and improve the remediation rate, using a combination of microbial remediation technology and other technologies such as electrokinetic remediation technology (Ma et al., 2018), photocatalytic remediation technology (Xu et al., 2017), nanotechnology (Alabresm et al., 2018) and bioreactor technology (Safdari et al., 2018) is an effective strategy to accelerate the removal of petroleum hydrocarbon pollutants.

## Conclusion and Future Outlook

Petroleum hydrocarbons are one of the most alarming pollutants due to their high toxicity to human and environmental health. Bioremediation with petroleum hydrocarbon-degrading bacteria is widely regarded as an eco-friendly and efficient technology. A large amount of bacterial species with petroleum hydrocarbon-degrading ability have been exploited and applied in bioremediation. However, various problems that slow down biodegradation effects have been found during the process of practical application. This review highlighted these restriction factors, including the toxic effects of petroleum hydrocarbons, the bioavailability of pollutants, environmental constraints, metabolic restrictions and time consumption, and then summarized the current countermeasures against these problems. Several strategies, such as regulating environmental factors and optimizing microbial inoculants, have been investigated and fulfilled. Based on the current state of knowledge reviewed here, a series of investigations still needs to be conducted prior to the successful application of bioremediation for the restoration of petroleum oil contaminated environments. It is concluded as follows: (1) Continue the theoretical basis of the interfacial interaction mechanism between bacteria and petroleum hydrocarbons in order to overcome barriers for microbial uptake of petroleum hydrocarbons, (2) develop novel biocompatible surfactants to enhance contact between bacteria and petroleum hydrocarbons, (3) explore undiscovered resources of petroleum hydrocarbon-degrading bacteria via new biotechnology, such as a high-throughput screening method to increase and enrich functional bacterial resources, (4) further optimize the strategy of artificial microbial consortia, such as by way of the metagenome enrichment approach to enrich and develop preferable consortia, (5) explore the novel functional genes controlling the pathway of hydrocarbon degradation to provide new looks on the molecular mechanism and microbial remediation, and (6) construct genetically engineered bacteria by using synthetic biology technology to give them more ability for petroleum hydrocarbon degradation.

## Author Contributions

XX and HY contributed to the writing of the manuscript. WL, ST, WW, QQ, PJ, XG, FL, and HL contributed to the collection of literatures and summarization.

## Conflict of Interest Statement

The authors declare that the research was conducted in the absence of any commercial or financial relationships that could be construed as a potential conflict of interest.
